# Commentary: EP-PINNs: Cardiac electrophysiology characterisation using physics-informed neural networks

**DOI:** 10.3389/fcvm.2022.1003652

**Published:** 2022-08-24

**Authors:** Stefan Meier, Jordi Heijman

**Affiliations:** Department of Cardiology, Cardiovascular Research Institute Maastricht (CARIM), Maastricht University, Maastricht, Netherlands

**Keywords:** atrial fibrillation, cardiac electrophysiology, computer model, machine learning, physics informed neural networks, parameter estimation

## Introduction

Atrial fibrillation (AF) is the most common cardiac arrhythmia with a significant medical and socioeconomic impact (1). Current AF management is centered around stroke-prevention, control of comorbidities and risk factors, and symptom management by rate- and rhythm control. Recent work has suggested that rhythm control may even improve clinical outcomes compared to rate control, particularly when initiated early, as demonstrated by the pivotal EAST AFNET-4 trial (2). Nevertheless, current options for rhythm-control therapy remain suboptimal due to the potential proarrhythmic side-effects of antiarrhythmic drugs (3) and limited efficacy of both drugs and catheter ablation, primarily attributable to a one-size-fits-most therapeutic approach (3, 4). A better understanding of the mechanisms underlying AF in an individual patient is expected to improve therapeutic success. However, identifying patient-specific mechanisms through cardiac mapping is challenging, invasive, time-consuming, and only gives partial information about the underlying electrophysiology under specific conditions (4). It is likely that novel approaches providing more detailed information on the underlying mechanisms will facilitate a more accurate localization of AF drivers, thereby enabling more personalized and better treatments.

Computer models offer perfect control and observability, facilitating a detailed investigation of arrhythmia mechanisms. In general, computer models can be divided into data-driven models and mechanistic models (3). Data-driven models use techniques like machine learning to identify complex relationships between datapoints to predict specific outcomes. For example, Attia et al. employed a neural network (NN) trained on a large dataset of electrocardiogram (ECG) recordings in sinus rhythm to identify patients with a diagnosis of AF (5). On the other hand, mechanistic models incorporate biophysical laws (typically as a system of differential equations) to simulate the dynamics of cardiac electrophysiology at the cellular-, tissue-, and/or organ-level (3). Mechanistic models have a long history and are increasingly being used in clinical and industry applications. For example, the “comprehensive *in-vitro* proarrhythmia assay” (CiPA) initiative from the pharmaceutical industry and regulatory agencies includes cellular mechanistic models to perform *in-silico* safety pharmacology screening for drug-induced ventricular arrythmias (3, 6). In addition, patient-specific organ-level models (“digital twins”) are starting to be used in clinical decision making. For example, Boyle et al. (7) used an organ-level modeling approach with patient-specific fibrotic patterns for preprocedural identification of AF ablation targets. The clinical value of such personalized simulation-guided AF ablation strategies is currently evaluated in a randomized clinical trial (ClinicalTrials.gov identifier NCT04101539).

Both mechanistic and data-driven modeling have pros and cons: data-driven models are, in theory, more easily applicable to clinical practice, since they use clinical parameters as inputs and outputs, and are hypothesis-free. However, the black-box nature of many algorithms hampers model interpretability and the models can only identify associations, which may not be causal. By contrast, mechanistic models are inherently causal and, in principle, could be personalized to reflect pathophysiology in an individual patient. However, at present, these models typically cannot predict clinically relevant outcomes. Furthermore, the tremendous computational power, together with the complex integration of imaging and cardiac mapping required for organ-level modeling, hampers its clinical application. Finally, both data-driven and mechanistic models require large amounts of (pre)clinical electrophysiological data from humans, which are challenging to obtain.

## EP-PINNs

**Figure 1:**
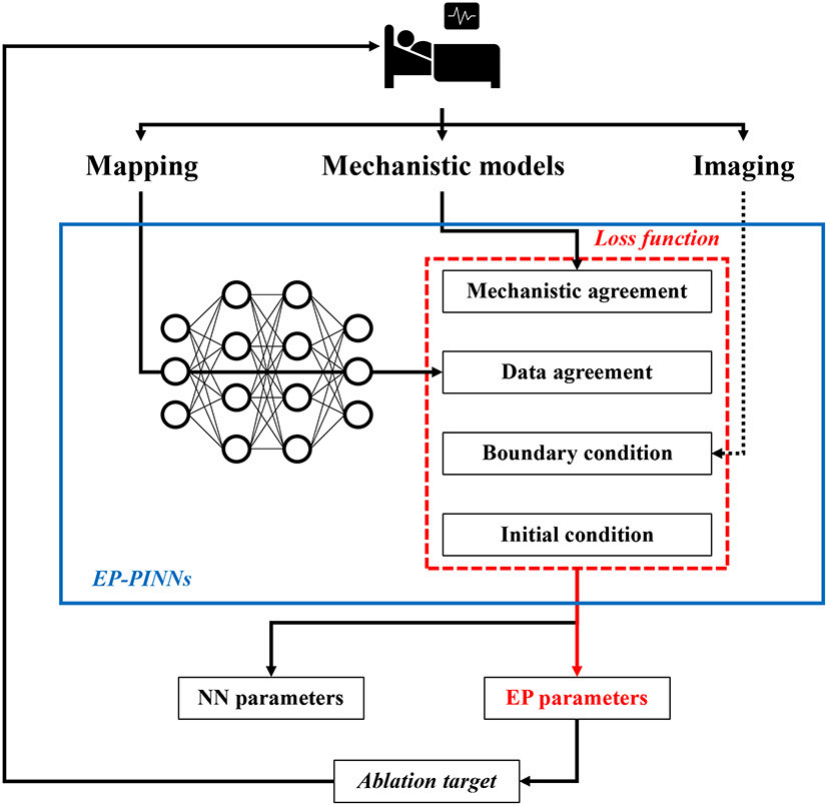
Potential future application of EP-PINNs. EP-PINNs use a neural network that integrates mechanistic models with its associated biophysical laws as constraints, while simultaneously fitting the observed mapping data. The estimated electrophysiological parameters can be used to identify ablation targets, thereby facilitating personalized therapy. *The dotted line highlights potential future additions of other data sources such as cardiac imaging.

Recently, a novel “hybrid” modeling approach, called physics-informed NNs (PINNs), has been proposed, which incorporates biophysical knowledge (i.e., mechanistic models of ordinary- and partial differential equations) to constrain the NN (4). In particular, PINNs are trained to minimize a loss function containing terms for the data mismatch, the concordance with physical laws, as well as initial and boundary conditions (Figure 1). The concordance with physical laws is defined as agreement with the differential equations of the model (i.e., the difference between the actual change in a model variable and its predicted change according to the differential equations). The training of this PINN requires fewer experimental data, since the solution space is constrained by the known relationships embedded in the differential equations, and ultimately provides parameters for both the NN and the mechanistic model, enabling identification of local properties captured by the mechanistic model. The latter could be particularly important for gaining a better understanding of the patient-specific mechanistical underpinnings of arrhythmias. In cardiac electrophysiology, PINNs have previously been used to estimate activation times and conduction velocities (CVs) to overcome the limited spatial resolution of clinical measurements, but the performance was suboptimal and could not easily be extended to other electrophysiological parameters (4, 8–10).

Recently, Herrero Martin et al. introduced “EP-PINNs” wherein the Aliev-Panfilov model of action-potential generation and propagation was used to constrain a NN fed with 2D simulation or experimental optical mapping data (4). The Aliev-Panfilov model uses two differential equations and six parameters to model action-potential propagation and refractoriness, enabling estimation of local electrophysiological parameters like action potential duration and CV. The EP-PINN was trained and evaluated on 1D and 2D *in-silico* data, whereafter it could reproduce a variety of spatio-temporal activation patterns (e.g., planar, centrifugal, and spiral waves). Proarrhythmic electrical heterogeneities (e.g., fibrosis, ischemic lesions) were incorporated by lowering the conductivity of a specific region. Importantly, the EP-PINN model could estimate the electrophysiological parameters (i.e., action-potential duration, excitability, and conductivity) related to these heterogeneities, albeit with lower accuracy than the homogeneous data, particularly in the presence of spiral waves. To further validate the findings from the *in-silico* data, the model's performance was evaluated on optical mapping data from neonatal rat ventricular cardiomyocytes in response to pharmacological interventions.

## Discussion

The main strengths of the EP-PINNs are the ability to predict, with varying accuracies, action-potential dynamics and parameters, even though the input data were noisy and sparse. Indeed, the *in-vitro* validation of the model shows that the model still performs well with noisy experimental data, although it required more training data. Moreover, the fact that the EP-PINNs performed well on a more complex canine atrial cardiomyocyte model suggests that the results are robust and that the model can be applied in a variety of settings. In the future, alternative, more complex mechanistic models could be integrated. Since the EP-PINNs approach provides estimates for model parameters that cannot be measured directly, these models may inform on specific remodeling processes. However, combined estimation of multiple parameters, particularly excitability combined with CV, proved challenging in the present study (4), highlighting potential difficulties for simultaneous parameter estimates in more complex models.

The use of biophysical laws for regularization also increased the computational efficiency tremendously, while still ensuring realistic behavior. Nonetheless, EP-PINNs remain complex and computationally expensive models due to their fully connected architecture and intricate training protocol, where higher-dimensional simulations will increase the training time substantially, particularly when more complex mechanistic models are going to be used.

Identification of spatial heterogeneities in underlying electrophysiological parameters, some of which cannot be measured directly experimentally, suggests that EP-PINNs could be used in the future to identify personalized ablation targets. However, in the study by Herrero Martin et al. (4) the EP-PINNs were trained on 2D spatio-temporal points from *in-silico* tissue simulations or optical mapping experiments, whereas the arrhythmogenic substrate in patients is 3D. It is not known whether EP-PINNs can resolve the complex 3D patterns of AF in clinical practice, which input data would be needed for this task, and what the computational requirements would be, although this could be investigated using available 3D mechanistic computational models. Moreover, the flexible structure of EP-PINNs in theory makes it possible to include additional constraints, e.g., based on mechanical or structural information from imaging data (Figure 1). Of note, for mechanical data, several mechanistic models operating on different scales are also available, which could be used in conjunction with electrophysiological models.

Taken together, Herrero Martin et al. showed that EP-PINNs are a potentially promising tool for integrating mechanistic modeling and machine learning. Although significant additional work is needed, EP-PINNs could ultimately aid personalized AF therapy by identifying local electrophysiological targets for ablation therapy based on patient-specific mapping and imaging data, combined with state-of-the-art understanding of AF pathophysiology captured by mechanistic models (Figure 1). The EP-PINNs' code is publicly available, which facilitates the first steps toward future clinical applications, e.g., by applying the EP-PINNs to more complex mechanistic models, simulate more chaotic arrhythmogenic patterns like multiple wavelets, and improve the computational efficiency of the model.

## Author contributions

All authors listed have made a substantial, direct, and intellectual contribution to the work and approved it for publication.

## Funding

This work was supported by the Netherlands Organization for Scientific Research (NWO/ZonMW Vidi 09150171910029 to JH).

## Conflict of interest

The authors declare that the research was conducted in the absence of any commercial or financial relationships that could be construed as a potential conflict of interest.

## Publisher's note

All claims expressed in this article are solely those of the authors and do not necessarily represent those of their affiliated organizations, or those of the publisher, the editors and the reviewers. Any product that may be evaluated in this article, or claim that may be made by its manufacturer, is not guaranteed or endorsed by the publisher.
